# Direct Care Staff Training Standards and Educational Interventions in Long-Term Care

**DOI:** 10.1093/geroni/igaf122.762

**Published:** 2025-12-31

**Authors:** Katherine Kennedy, Carissa Coleman

## Abstract

The training of direct care staff including personal care aides (PCAs), nursing assistants (NAs), and student nurses is critically important to delivery of high-quality care and a more stable long-term care workforce. Relevant quality training is key to ensuring optimum care and coordination for patient populations facing a spectrum of needs, such as those with dementia, mental illness, and other physical and cognitive impairments. Positive perceptions of training quality and attributes (e.g., greater hours, clinical training, facilitator, length, modality, content, impact on interprofessional practice) are associated with more positive perceptions of dementia, improved job satisfaction, and better nursing home resident health outcomes. While training contributes to job quality, current evaluations of training standards, actual training opportunities, and educational interventions or programs have been lacking in the field. A primary focus of this symposium is to describe the importance of workforce development in the post-pandemic world from a macro-policy lens focused on training consistency, rigor, and portability, to a micro-level focus on areas of opportunity for developing and implementing training (e.g., increasing job relevant training, enhancing digital literacy, microlearning). Presentations will highlight current information about training standards for PCAs, on-the-job training of nursing home staff (primarily NAs), and an innovative research-practice partnership to build a pipeline for student nurses into nursing home careers. Through examinations of training standards, training opportunities, and intervention/program delivery, we gain knowledge about potential practice gaps such as important knowledge to mitigate care challenges, knowledge translation strategies, and opportunities for program and policy development.

